# Editorial: Data governance in African health research: ELSI challenges and solutions

**DOI:** 10.3389/fphar.2024.1525434

**Published:** 2024-12-11

**Authors:** Donrich Thaldar

**Affiliations:** University of KwaZulu-Natal, Durban, South Africa

**Keywords:** consent, health research, data governance, open science, common heritage of humanity, genomic sovereignty, pseudonymised data, AI

This Research Topic, *Data Governance in African Health Research: ELSI Challenges and Solutions*, brings together analyses that address the emerging legal, ethical, and social issues surrounding data governance in African health research. As health research in Africa increasingly utilises digital data, artificial intelligence (AI), and genomic technologies, these articles explore the path forward, offering practical and legal insights into how Africa’s unique challenges can be addressed. Together, these contributions set out a forward-looking vision, guiding data governance toward a framework that respects participant rights, aligns with African regulatory environments, and adapts to evolving technological and ethical demands.

## Legal and ethical frameworks for consent: empowering research participants

At the heart of data governance lies the principle of consent, which is both a legal requirement and an ethical commitment to participant autonomy. In *Introducing Dynamic Consent for Improved Trust and Privacy in Research Involving Human Biological Material and Associated Data in South Africa*, Prinsen advocates for dynamic consent, a model that aligns with South Africa’s Protection of Personal Information Act 4 of 2013 (POPIA) by granting participants ongoing control over their data. This approach recognises the dynamic nature of health data use, positioning participants as active decision-makers, which strengthens both legal compliance and public trust.

Naidoo’s *Open Optimism as an* “*Embodied-Health*” *Ethic for the Information Era* offers a complementary vision, presenting an “embodied” ethical approach to health data that challenges traditional divides in health governance. By valuing openness and participant engagement, Naidoo highlights the role of ethics in reinforcing participant agency and enhancing transparency in African health research. Together, Prinsen and Naidoo’s contributions envision an African health research environment where ethical frameworks are responsive, participatory, and empower individuals while upholding shared values.

## Legal instruments and data management: establishing robust governance for health research

A clear legal and data management framework is crucial for protecting privacy and upholding ethical standards in health research. This Research Topic includes several articles addressing these aspects, presenting actionable insights for researchers, institutions, and policymakers. *The Anatomy of a Data Transfer Agreement for Health Research* by Swales et al. provides a comprehensive guide to creating data transfer agreements (DTAs) that align with data protection legislation, such as POPIA. This article underscores the importance of detailed DTA provisions that protect both data privacy and the legal interests of institutions when sharing sensitive research data. The research presented in this article formed the foundation for a freely accessible DTA template that was developed for the South African research community (Swales et al., 2023; Thaldar et al., 2024a; Thaldar et al., 2024b).

Building on the theme of legal compliance, *A Data Management Plan for the NESHIE Observational Study* by Strydom et al. offers a template for constructing data management plans (DMPs) that address the lifecycle of sensitive health data, including security, storage, and access considerations. This template is especially relevant for studies in low- and middle-income countries (LMICs), highlighting ways to balance the complexity of data management with rigorous compliance measures. By adapting this DMP framework, institutions can establish clear standards that both protect participant privacy and meet legal obligations.

Adding further depth to the discussion, *Forcing a Square into a Circle: Why South Africa’s Draft Revised Material Transfer Agreement is Not Fit for Purpose* by Esselaar et al. critiques South Africa’s National Health Research Ethics Council’s (NHREC) draft revision of South Africa’s standard material transfer agreement (MTA). The authors draw on the foundational work done by Thaldar et al. (2022) that explored the various legal dimensions of genetic data under South African law—including privacy, ownership, and intellectual property rights—but go further by advocating for a decolonial approach to health research governance, urging the NHREC to empower local research institutions by acknowledging their ownership of the data that they collect and generate.

## Open science: transparency and access in genomic research

Open science principles in genomic research promote accessible scientific knowledge and equitable benefit-sharing. In *A Pathway to Strengthening Open Science: Comments on the Draft South African Ethics in Health Research Guidelines*, Gooden critiques South Africa’s NHREC draft South African Ethics in Health Research Guidelines, advocating for ethics guidelines that incorporate open science principles and promote African-centric approaches to enhance transparency and legal compliance in African health research. Gooden’s recommendations align with *Open Science and Human Genetic Data: Recommendations on South Africa’s Draft National Open Science Policy*, where Thaldar et al. underscore the importance of the right to freedom of scientific research, the legal difference between human and non-human genetic data, and data ownership. Importantly, open science does not require data to become public property. Instead, data can remain private property, allowing data originators to benefit while fostering responsible sharing.

## Common heritage vs. genomic sovereignty: competing frameworks in genomic research

In *The Human Genome as the Common Heritage of Humanity* (Kabata and Thaldar) and *Regulating Human Genomic Research in Africa: Why a Human Rights Approach Is a More Promising Conceptual Framework than Genomic Sovereignty* (Kabata and Thaldar) examine two approaches to human genomic data as forms of public property, highlighting their practical and ethical implications.

The “common heritage” model views the human genome—often represented by the human reference genome—as a shared asset that belongs to all of humanity. This concept, grounded in international human rights, aims to protect genomic data from privatisation by framing it as an international public good, freely accessible for scientific advancement and collaboration. The focus is on global inclusivity, with genomic resources managed for the collective benefit of humanity.

In contrast, the genomic sovereignty model shifts from a global perspective to a national or community-based one, claiming that genomic data is the exclusive property of specific groups or nations. This approach, driven by concerns over resource exploitation and national interests, empowers countries or population groups to assert control over their genetic resources, restricting external access to protect local interests. The genomic sovereignty model, however, has been criticised for limiting international collaboration.

Kabata and Thaldar propose that a human rights-based framework offers a more balanced and ethical pathway. This approach respects individuals’ rights to benefit from scientific advancements while allowing for private ownership of genomic data.

## Foundational concepts in data law: considering pseudonymised data

In *Does Data Protection Law in South Africa Apply to Pseudonymised Data?*, Thaldar examines whether pseudonymised datasets fall under POPIA in South Africa, arguing that identifiability—and therefore POPIA’s applicability—depends on the specific context of the party handling the data. By interpreting POPIA’s exclusions clause and research exception through established South African legal principles, Thaldar concludes that identifiability should be assessed contextually: A dataset remains personal information for a provider retaining both the pseudonymised and linking datasets, but becomes non-personal for a recipient without access to linking data. This approach balances privacy protection with data-sharing flexibility, enabling responsible, context-sensitive data management in health research. Thaldar’s insights are particularly valuable as South African institutions navigate complex privacy demands, highlighting the need for legal clarity in an evolving research environment.

## AI as the new frontier: defining agency and liability in health research

AI is advancing rapidly in health research, presenting new legal and ethical challenges. *Mapping the Regulatory Landscape of AI in Healthcare in Africa* by Townsend et al. survey AI regulations across 12 African countries, identifying regulatory gaps and recommending cohesive frameworks that can support ethical AI adoption. Townsend et al. highlight that Africa must develop robust AI governance to balance innovation with protection for participants, establishing a regulatory foundation for the continent’s AI future.

*Liability for Harm Caused by AI in Healthcare: An Overview of the Core Legal Concepts* by Bottomley and Thaldar, explores liability Research Topic specific to AI in healthcare, considering legal approaches such as strict liability and the principal–agent relationship. These frameworks aim to clarify accountability in cases where AI systems cause harm, underscoring the need for legal structures that can address AI’s unique risks.

Adding depth to AI governance, Naidoo’s *What Does It Mean to Be an Agent?* proposes a practical framework for assessing AI agency, focusing on empirical characteristics rather than abstract notions like consciousness. This grading system provides a structured, adaptable model for regulating AI, considering both legal accountability and suitability for specific research contexts. Naidoo’s *The Open Ontology and Information Society* further frames AI governance within a broad ethical and legal structure, proposing a qualitative analysis of information to inform regulatory approaches. These articles lay a groundwork for legally and ethically responsible AI governance in African healthcare, ensuring that AI serves the public good within a framework of accountability and participant protection.

## Building an African framework for health research governance

Together, these articles provide a comprehensive guide to advancing data governance in African health research. Each contribution demonstrates a commitment to addressing Africa’s unique challenges, from privacy laws and consent frameworks to policy guidance and AI governance. This Research Topic offers insights that will help shape Africa’s health research landscape in a way that respects individual rights, supports responsible innovation, and aligns with evolving ethical and legal standards. The future of data governance in African health research is a complex, rapidly evolving field, but with a foundation rooted in law and ethics, African researchers and policymakers are well-positioned to navigate it with confidence.
